# Co-existence of Rhizobia and Diverse Non-rhizobial Bacteria in the Rhizosphere and Nodules of *Dalbergia odorifera* Seedlings Inoculated with *Bradyrhizobium elkanii, Rhizobium multihospitium*–Like and *Burkholderia pyrrocinia–*Like Strains

**DOI:** 10.3389/fmicb.2017.02255

**Published:** 2017-11-21

**Authors:** Junkun Lu, Fucheng Yang, Shengkun Wang, Haibin Ma, Junfeng Liang, Yinglong Chen

**Affiliations:** ^1^State Key Laboratory of Tree Genetics and Breeding, Research Institute of Tropical Forestry, Chinese Academy of Forestry, Guangzhou, China; ^2^State Key Laboratory of Soil Erosion and Dryland Farming on the Loess Plateau, Northwest A&F University, Yangling, China; ^3^Institute of Soil and Water Conservation, Chinese Academy of Sciences, Yangling, China; ^4^Institute of Agriculture, and School of Agriculture and Environment, The University of Western Australia, Perth, WA, Australia

**Keywords:** bacterial communities, *Dalbergia odorifera*, high-throughput sequencing, nitrogen fixation, non-rhizobial bacteria, rhizobia

## Abstract

Rhizobia induce root nodules and fix atmospheric N_2_ for most legume species in exchange for carbon. However, the diverse endophytic non-rhizobial bacteria in legume nodules that co-exist with rhizobia are often ignored because they are difficult to cultivate using routine cultivation approaches. To enhance our understanding of the incidence and diversity of legume–bacteria associations, a high-throughput sequencing analysis of bacterial 16S rRNA genes was used to examine the bacterial community in the rhizospheres and root nodules of *Dalbergia odorifera* seedlings that were uninoculated or inoculated with *Bradyrhizobium elkanii* H255, *Rhizobium multihospitium–*like HT221, or *Burkholderia pyrrocinia–*like H022238, in two growth media (nitrogen [N]-supplied soil or N-omitted potting mix). Seedlings inoculated with *Bradyrhizobium* had significantly more nodules than seedlings in the other inoculation conditions, regardless of growth media. Using the ^15^N natural abundance method, it was shown that the inoculated plants had significantly higher N_2_ fixation efficiency (48–57%) and specific nodule activity [269–313 μg N mg^−1^ of dry weight (dwt) nodule] compared to the uninoculated plants (203 μg N mg^−1^ dwt nodule). The 16S rRNA gene analysis showed that there was generally a higher bacterial diversity in the rhizosphere than in the nodules in the corresponding condition. Both rhizobial inoculation and media status significantly altered the bacterial communities in the rhizospheres and nodules (*P* < 0.05), with the exception of the inoculated soil rhizospheres. Regarding non-rhizobial bacteria, three genera, i.e., *Lactococcus, Bacillus*, and *Pseudomonas*, were consistently enriched in the rhizosphere and *Bradyrhizobium, Chloroplast* norank (which belongs to *Cyanobacteria*), and *Lactococcus* were commonly found in the nodules. In contrast, common rhizobial genera (including *Rhizobium, Mesorhizobium*, and *Burkholderia*) were only present in the nodules at low relative abundances (0.01–3.41%). Regarding non-rhizobial bacteria, 32 genera were found in the nodules, with non-rhizobial bacteria being predominant in the N omitted potting mix (with a relative abundance of 56–87%). This study suggests that legume nodules are inhabited by a high diversity of non-rhizobial bacteria, which may play a vital role in nodulation and N_2_ fixation in the host plants.

## Introduction

Plants grow in intimate association with heterotrophic microorganisms, and they provide carbohydrates for the growth of these microorganisms. Reciprocally, some types of microbes [including free-living nitrogen (N_2_) fixers, intercellular endophytic bacteria, and symbiotic bacteria] are able to fix atmospheric N_2_ in exchange for carbon from their plant hosts (Mus et al., 2016; Sprent et al., 2017). To adapt to this habitat, a limited range of microbes, e.g., endophytes and symbionts, have evolved the ability to colonize external or internal plant tissues with little or no host damage (Bulgarelli et al., 2013).

The N_2_-fixing symbiosis between most legumes and bacteria is a well-studied example of the formation of root nodules, and occasionally stem nodules, which are induced and subsequently invaded by specific rhizobia. Phylogenetic studies have shown that, based on a diverse range of legume species, over 150 rhizobial species belonging to 12 genera of *Alphaproteobacteria* and two genera of *Betaprotebacteria* occupied the root nodules tested (Peix et al., 2015; Zahran, 2017). In various legumes, in addition to rhizobia that are responsible for nodulation and N_2_ fixation, other endophytic bacteria, called non-rhizobial bacteria, are also found in the nodules (Sachs and Simms, 2008; Wu et al., 2011; Busby et al., 2016). For example, 71 genera, including *Dyella, Enterobacter, Pseudomonas*, and *Steroidobacter*, were observed in *Lespedeza* nodules (Busby et al., 2016); eight genera comprising *Paenibacillus, Bacillus, Klebsiella, Ensifer, Agrobacterium, Blastobacter, Dyadobacter*, and *Chitinophaga* were isolated from the nodules of field-grown *Vigna radiate* (Pandya et al., 2013); and a wide variety of non-rhizobial bacteria (e.g., *Agrobacterium, Enterobacter, Paenibacillus*, and *Phyllobacterium*) can colonize *Glycyrrhiza* nodules (Li et al., 2012). Some of these non-rhizobial bacteria have proven beneficial to their legume hosts, as they enhance plant growth by producing plant hormones, fixing atmospheric N_2_, and solubilizing phosphate (Peix et al., 2015). Nevertheless, these non-rhizobial bacteria have been disregarded in the past during the isolation of rhizobia from legumes [using the traditional cultivation method involving the use of yeast-mannitol-based agar (YMA)]. This led to a lack of information on the true diversity of non-rhizobial bacteria coexisting with rhizobia in the nodules, and on their possible essential roles in nodulation and N_2_ fixation. Current metagenomic approaches can be used to shed light on the situation by allowing the detection of both rhizobia and non-rhizobial bacteria in nodules. This approach may help to accurately determine the roles of bacterial species in the nodulation and N_2_ fixation processes.

*Dalbergia odorifera* T. Chen is one of the extremely precious rosewoods that are native to Southern China and that have both medicinal and commercial value (Ma et al., 2013; Lee et al., 2014). When it is effectively fixing N_2_, *D. odorifera* has been shown to be a suitable host for the hemiparasite *Santalum album* (Indian sandalwood) (Lu et al., 2013, 2014). Unlike other species of *Dalbergia, D. odorifera* is distinguished by its capacity to form N_2_-fixing nodules after the invasion of rhizobia (Gao et al., 1994; Rasolomampianina et al., 2005; Lu et al., 2011, 2012). We recently isolated a number of bacterial strains (belonging to *Agrobacterium, Bradyrhizobium, Ensifer, Rhizobium*, and *Burkholderia*) from *D. odorifera* nodules obtained from various locations in Southern China (Lu et al., 2011, 2012). Identification of bacteria in nodules has traditionally relied on their cultivability when streaked on YMA plates. Thus, in the past, complete information on the diversity of uncultured rhizobia and non-rhizobial bacteria was not obtained.

This study used three strains of rhizobia (originally isolated from *D. odorifera*) to inoculate *D. odorifera* seedlings, and the effect of the inoculation on host performance and N_2_ fixation was assessed. We hypothesized that (i) inoculation influences host N_2_ fixation; (ii) inoculation has a significant impact on bacterial communities in both the rhizosphere and nodules; and (iii) non-rhizobial bacteria are relatively abundant compared to rhizobia. The outcomes of this study could improve our understanding of legume–bacteria associations.

## Materials and methods

### Experimental design

A randomized split-plot design was used, which involved four inoculation conditions (non-inoculation and inoculation with three rhizobial strains) as the first factor and two growth media [N-supplied soil (N+) and N-omitted potting mix (N−)] as the second factor. For each treatment combination, six replicates (plants) were assessed in a greenhouse study.

### Rhizobia strains and inoculum preparation

Three rhizobial strains, *Bradyrhizobium elkanii* (H255), *Rhizobium multihospitium*–like (HT221), or *Burkholderia pyrrocinia*–like (H022238), isolated from the root nodules of *D. odorifera* obtained from various geographic locations in China, were used in this inoculation experiment (Table 1). The GenBank accession numbers for the 16S rRNA, *recA*, and *nodC* gene sequences are listed in Table 1. Phylogenetic analysis of the 16S rRNA gene showed that strains H255, HT221, and H022238 were most closely related to *Bradyrhizobium elkanii* USDA76^T^ (100%), *Rhizobium miluonense* CCBAU41251^T^ (99.2%) and *Burkholderia pyrrocinia* JK-SH007 (98.4%), respectively (Figure S1).

**Table 1:** The three strains used in this study.

**Strains**	**Isolation sites**	**16S rRNA**	***recA***	***nodC***
*Bradyrhizobium elkanii* H255	Jiangfeng, Hainan Province, China	KX159762	KX159756	KX159752
*Rhizobium multihospitium* –like HT221	Pingxiang City, Guangxi Province, China	KX159767	KX159761	–
*Burkholderia pyrrocinia*–like H022238	Sanya City, Hainan Province, China	KX159766	KX159760	KX159755^*^

The amplifying primers and conditions of 16S rRNA, recA, and nodC were described by DeLong (1992), Vinuesa et al. (2005), and Laguerre et al. (2001), respectively. –, not detected.

* *The nodC gene of strain H022238 shared the highest similarity (99.6%) with the Bradyrhizobium sp. CCBAU 51595 (KF114578)*.

Bacterial suspensions (10^7^-10^9^ cfu/ml in sterilized 0.75% NaCl) of each of the three strains cultivated for 3–5 days on yeast-mannitol media in 500 ml triangular flasks at 28°C (centrifuged at 4,000 rpm, 5 min) were used as the rhizobia inocula in this study.

### Planting, inoculation, and fertilization

The experiment was carried out in a temperature-controlled glasshouse at the Research Institute of Tropical Forestry, Chinese Academy of Forestry, Guangzhou, China (23°11′N, 113°23′E), with day/night temperatures of 31/23°C and a relative humidity of 68/80%. Seeds of *D. odorifera* were surface-sterilized with 3% sodium hypochlorite for 5 min, and then washed three times with distilled water. The seeds were then incubated on sterile moistened filter paper in Petri dishes at 28°C in the dark until germination (~3 weeks).

Germinated *D. odorifera* seedlings with 2-cm radicals were soaked in the rhizobia preparations for 5 min before transplanting them into pots. A further 2 ml/pot of the rhizobia inoculum was placed around the taproot area 3 days after transplantation. Regarding the uninoculated condition, the seedlings were administered the same quantity of sterilized 0.75% NaCl solution.

Two media conditions were used to study N_2_ fixation efficiency and to compare the bacterial communities in different growth media conditions. The seedlings were transplanted into plastic pots (maximum diameter, 14 cm, depth, 12 cm). For the N+ condition, the pots were filled with 1 kg field soil (Utisol) that was collected from a forest within the grounds of the Research Institute of Tropical Forestry (the soil contained 5.16 g·kg^−1^ organic matter, 0.30 g·kg^−1^ total N, 0.15 g·kg^−1^ total P, and 7.19 g·kg^−1^ total K, and it had a pH of 4.5 in deionized water). For the N− condition, the pots were filled with 250 g potting mix (vermiculite: perlite, 2:1, v/v, pH 6.5), which was organic-matter free and had almost no N.

The plants were grown for 8 months (December 2013 to August 2014). Each pot was watered three times per week with tap water and fertilized monthly with 50 ml N-free Jensen nutrient solution (Burdon et al., 1999). For the N+ condition, 1 ml 0.5% (NH_4_)_2_SO_4_ solution was supplied monthly.

The N_2_ fixation efficiency was calculated using Equation 1 (Knowles and Blackburn, 1993):

(1) $$\%{\text{N}}_{\text{fixed}}=\frac{\left(\delta^{15}{\text{N}}_{\text{non }-\text{ fixing plant}}-\delta^{15}{\text{N}}_{\text{fixing plant}}\right)}{\left(\delta^{15}{\text{N}}_{\text{non }-\text{ fixing plant}}-\text{B}\right)}\times 100$$

where B is the δ^15^N value of the nodulated N_2_-fixing plants grown under N-free conditions. Two local indigenous woody plants, the first being *Bischofia polycarpa* (Levl.) Airy Shaw and the second being *Michelia macclurei* Dandy, were chosen as the non-N_2_-fixing reference plants to calculate the averaged foliar δ^15^N values for the equation.

### Plant harvesting and sampling

Eight months after transplantation, the leaves and nodules of *D. odorifera*, along with the rhizosphere soils, were collected. The plant leaves were oven-dried (70°C for 72 h) and ground in a ball mill (Retsch GmbH & Co. KG, Haan, Germany) to analyze the total N and δ^15^N using a spectrometer (Isotope Ratio Mass Spectrometer, Thermo Fisher Scientific Inc., Waltham, MA, USA).

For each treatment combination, fresh nodules (0.5 g) from three plant replicates were surface sterilized (in 95% ethanol for 45 s followed by immersion in 1% HgCl_2_ for 3 min), washed with sterile water, and then stored at −80°C until DNA extraction.

### DNA extraction

For each treatment combination, the total genomic DNA was extracted from 0.5 g of nodule and rhizosphere samples from three plant replicates using an E.Z.N.A. soil DNA kit (Omega Biotek, Norcross, GA, USA) following the manufacturer's protocol. The presence of DNA was evaluated by carrying out electrophoresis on 1% agarose gel (Biowest, Barcelona, Spain) with a 1-kb DNA ladder (Pomega, Madison, WI, USA). The DNA concentration was determined using a spectrophotometer (Nanodrop 2000, Thermo Fisher Scientific), and samples with values >0.1 μg/μl were used in subsequent analyses. Extracted DNA were used as templates for subsequent PCR. As there were eight treatment combinations and two replicates per treatment combination, there were 16 rhizosphere and 16 nodule samples, comprising a total of 32 samples.

### PCR amplification

The DNA was PCR-amplified using universal bacterial primers: 515F (5′-GTGCCAGCMGCCGCGG-3′) and 907R (5′-CCGTCAATTCMTTTRAGTTT-3′). These primers were selected to amplify the V4+V5 variable region of the 16S rRNA gene in the bacteria, creating an amplicon of ~390 bp.

Each PCR mixture contained 0.8 μl of each primer (5 μM), 2 μl 2.5 mM deoxynucleotide triphosphates (dNTPs; BBI, Ontario, Canada), 0.4 μl FastPfu polymerase (TransGen Biotech, Beijing, China), 10 ng genomic DNA, and 4 μl 5 × FastPfu Buffer (TransGen Biotech, Beijing, China), with a final reaction volume of 20 μl. The amplifications were carried out using GeneAmp 9700 PCR (Applied Biosystems, Carlsbad, CA, USA) using the following program: 95°C for 3 min, then 27 cycles of 95°C for 30 s, 55°C for 30 s, and 72°C for 45 s, ending with 72°C for 10 min.

### Illumina MiSeq sequencing

The amplicons were extracted using 2% agarose gels and purified using an AxyPrep DNA Gel Extraction Kit (Axygen Biosciences, Union City, CA, USA) according to the manufacturer's instructions, and they were then quantified using QuantiFluor™-ST (Promega, Madison, WI, USA). The purified amplicons were pooled in equimolar quantities and paired-end sequenced (2 × 250) using an Illumina MiSeq platform according to the standard protocols. The raw reads were deposited in the National Center for Biotechnology Information (NCBI) Sequence Read Archive database (BioProject ID: PRJNA322091).

### Processing of sequencing data

All the sequence reads were checked for quality. Using QIIME (version 1.17), poor-quality reads and primer dimers were discarded. Using Usearch (version 7.1), operational taxonomic unit (OTU) clustering was performed, with a 97% similarity threshold. Singletons and chimeric sequences were filtered out. The taxonomy of each 16S rRNA gene sequence was analyzed using the RDP Classifier (http://rdp.cme.msu.edu/) against the SILVA (SSU115) 16S rRNA database, based on a confidence threshold of 70% (Amato et al., 2013). To reduce the influence of sequencing depth on the estimated effect sizes, a subsample of 10,461 sequences was selected for each sample.

### Statistical analysis

The effects of the rhizobial inoculation and media conditions were assessed using two-way analysis of variance (ANOVA). Where the effects were significant, a Tukey–Kramer honestly significant difference (HSD) test was used for pairwise comparisons of the means. One-way ANOVA was used to compare the effects of rhizobial inoculation on N_2_ fixation efficiency and specific nodule activity. A *P* < 0.05 was considered significant. The data were analyzed using PASW Statistics software version 18 (SPSS Inc., Chicago, IL, USA).

Using QIIME, an Adonis analysis based on Bray–Curtis dissimilarity was used to evaluate the significance of the differences in the bacterial communities between the treatment combinations and between each pair of samples in a given treatment combination group. For each treatment combination, the mean number of OTUs of the two replicates was used for the subsequent bioinformatic analyses. Diversity indexes [abundance-based coverage estimator (ACE), Chao1, and the Shannon index] were assessed using MOTHUR. The relative bacterial abundances and a cluster tree were generated, and a principal component analysis (PCA) was performed, using the R statistical platform (version 3.2.4).

## Results

### N_2_ fixation capacity of *D. odorifera*

**Figure 1:**
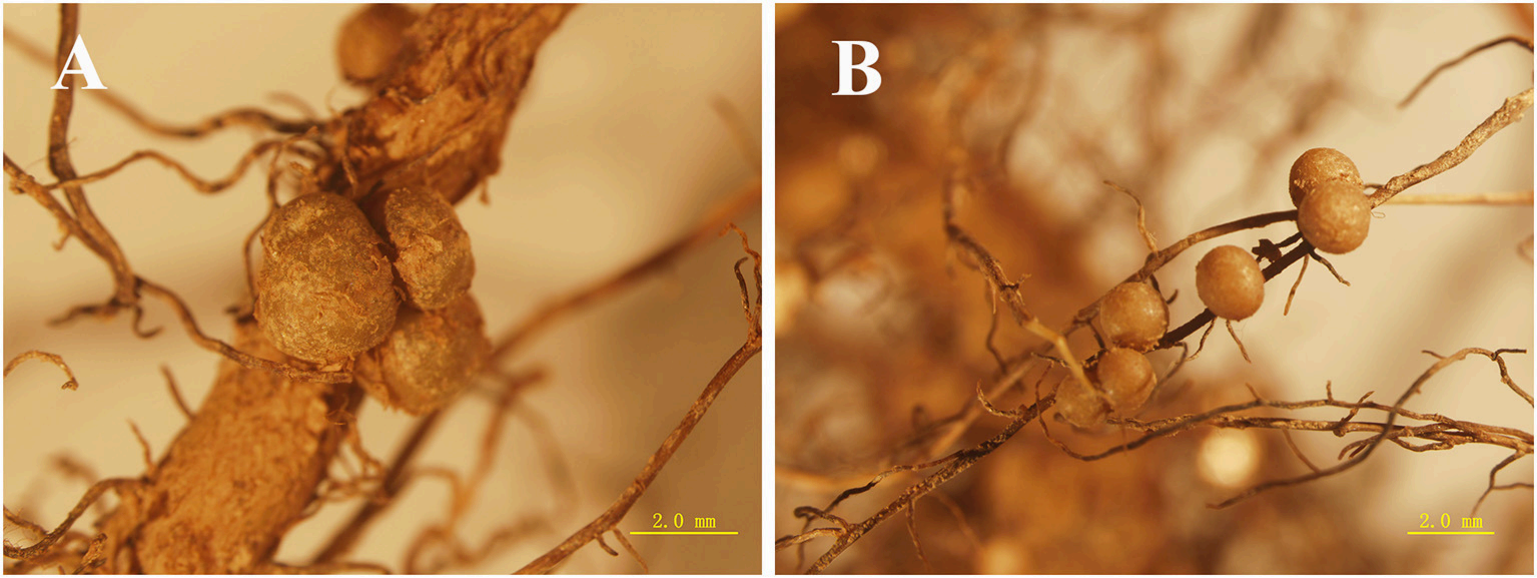
Nodules formed on the axial **(A)** and lateral **(B)** roots of *D. odorifera* seedlings.

In both media conditions, reddish-brown nodules were observed on the roots of *D. odorifera* (Figure 1). In general, significantly more nodules were observed on N− seedlings than on the N+ seedlings (*P* < 0.01) (Table S1), with the exception of those in the *Burkholderia* inoculation condition (Table 2). Irrespective of media status, *D. odorifera* inoculated with *Bradyrhizobium* had significantly more nodules than the plants in the other inoculation conditions (*P* < 0.01). Plants inoculated with *Burkholderia* had significantly lower foliar δ^15^N values than those in the other inoculation conditions, regardless of media status (*P* < 0.01).

**Table 2:** N_2_-fixation capacity of 8-month-old *D. odorifera* seedlings grown in pots.

**Treatments**	**Nodule numbers**		**Foliar** δ^**15**^**N (%0)**		**N concentration (%)**		**N_fixed_ (%)**	**Specific nodule activity (μg N mg^−1^ dwt nodule)**
	**N+**	**N−**	**N+**	**N−**	**N+**	**N−**		
*Bradyrhizobium elkanii* H255	9 ± 1 a, y	129 ± 10 a, x	−1.37 ± 0.05 a, x	−1.92 ± 0.10 a, y	3.40 ± 0.12 a, x	2.21 ± 0.19 a, y	57 ± 1 a	313 ± 13 a
*Rhizobium multihospitium*-like HT221	4 ± 1 b, y	45 ± 8 b, x	−1.41 ± 0.09 a, x	−2.15 ± 0.17 a, y	3.42 ± 0.15 a, x	1.94 ± 0.15 a, y	51 ± 4 ab	287 ± 26 a
*Burkholderia pyrrocinia*–like	6 ± 1 b, x	8 ± 1 c, x	−2.69 ± 0.34 b, x	−4.81 ± 0.57 b, y	3.93 ± 0.24 a, x	2.02 ± 0.24 a, y	48 ± 4 ab	269 ± 32 ab
No inoculation	4 ± 1 b, y	32 ± 3 bc, x	−1.33 ± 0.03 a, x	−2.15 ± 0.09 a, y	3.80 ± 0.16 a, x	2.04 ± 0.16 a, y	45 ± 1 c	203 ± 13 c

*Mean data (n = 6) of each media treatment (in column) followed by the same letter (a, b, or c) are not significantly different among the inoculation treatments (P < 0.05); Mean data of each inoculation treatment (in line) followed by the same letter (x or y) are not significantly different among the media treatments (P < 0.05). For the plants used for the N_2_-fixation measurements, foliar δ^15^N values were −0.61 ± 0.08 and −0.71 ± 0.21 for non-N_2_-fixing B. polycarpa and Michelia macclurei, respectively. N_2_-fixation was calculated by Eq. (1) using the averaged foliar δ^15^N values of these two non-N_2_-fixing plants*.

For each media condition, the tissue N concentrations were similar between inoculation conditions (Table 2). The N+ seedlings always had a higher tissue N concentration than the N− seedlings. Among the plants in the four inoculation conditions, N_2_ fixation supplied 45–57% of the total N requirement, and the specific nodule activity was 203–313 μg N mg^−1^ of dry weight (dwt) nodule (Table 2). Significantly higher N_2_ fixation efficiency and specific nodule activity were observed in the inoculated plants compared to the uninoculated plants (*P* < 0.05) (Table S2).

### Sequencing and quality control

The 32 samples collected from the rhizospheres and nodules provided a total of 878,192 raw reads. After quality filtering and chimera removal, 854,805 reads remained to be analyzed, with a mean length of 248 bp. The number of sequences per sample varied from 14,871 to 39,015. Using a 97% sequence similarity threshold, 2,949 bacterial OTUs were identified after randomly resampling the 16S rRNA gene sequence reads to the same depth (10,461 sequences per sample). However, this sequencing depth was not sufficient to assess the vast diversity of the bacterial communities in this study (Figure S2). The 32 samples exhibited a clear and significant (*P* = 0.001, Adonis) separation. The OTU data on the nodules differed significantly (*P* = 0.01–0.03, Adonis) from the data on the rhizospheres, regardless of the inoculation or media conditions. Rhizobial inoculation significantly influenced the nodule and rhizosphere bacterial communities (*P* = 0.01–0.04, Adonis), except in the N+ rhizosphere samples (*P* = 0.10, Adonis).

### Diversity indices

The ACE and Chao1 richness estimators, Goods' coverage, and Shannon index were calculated based on a 3% genetic distance for each sample (Table 3). The Chao1 estimator revealed a significant difference in bacterial community richness between the rhizosphere and nodule samples. Indeed, the estimated number of OTUs was 1.90–2.97 times lower in nodules (with means of 322 and 405 OTUs for the N+ and N− nodules, respectively) than in the corresponding rhizospheres (with means of 899 and 703 OTUs for the N+ and N− rhizospheres, respectively). The rhizosphere diversity indices (ACE, Chao1, and Shannon index) were generally higher than those for the nodule samples, with one exception: the N+ nodules in the uninoculated condition had a similar Shannon index to the corresponding rhizospheres. Rhizobial inoculation showed no clear pattern regarding bacterial community richness.

**Table 3:** OTU richness and diversity indices using a subset of sequences per treatment.

**Treatments**	**OTUs**	**ACE**	**Chao**	**Shannon index**	**Coverage (%)**
***Bradyrhizobium elkanii*** **H255**					
N+ rhizosphere	771	2,692	1,586	3.02	95.6
N+ nodule	250	1,339	645	1.71	98.5
N− rhizosphere	716	2,663	1,490	3.52	95.9
N− nodule	247	890	573	1.47	98.6
***Rhizobium multihospitium*****-like HT221**					
N+ rhizosphere	1,114	1,613	1,526	4.70	95.9
N+ nodule	267	1,220	768	1.77	98.4
N− rhizosphere	697	2,674	1,632	3.40	95.9
N− nodule	569	1,557	1,147	3.30	97.2
***Burkholderia pyrrocinia*****-like H022238**					
N+ rhizosphere	890	2,580	1,724	3.35	95.2
N+ nodule	345	1,126	793	1.64	98.1
N− rhizosphere	844	2,047	1,603	3.75	95.7
N− nodule	423	1,656	1,036	2.59	97.6
**No inoculation**					
N+ rhizosphere	819	2,584	1,642	3.26	95.3
N+ nodule	439	1,049	777	3.26	98.0
N− rhizosphere	555	2,752	1,362	3.15	96.5
N− nodule	379	1,100	742	2.50	98.1

*Data were obtained via analyzing the mean OTU value of two replicates of each treatment*.

### Taxa distribution in the 32 samples

**Figure 2:**
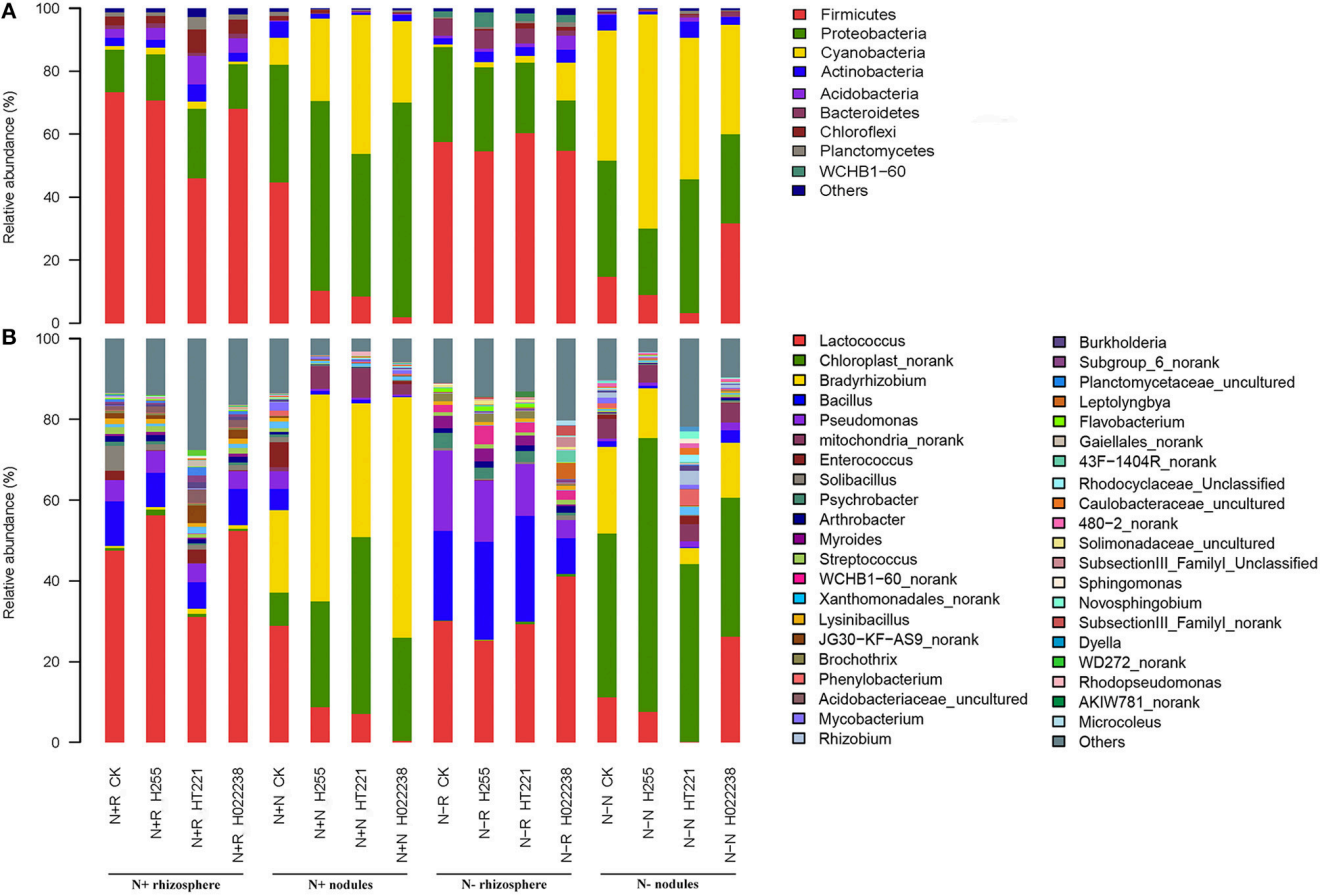
Relative abundance of the dominant phyla **(A)** and genera **(B)** in the rhizosphere and nodules of *D. odorifera* seedlings. N + R and N–R: *D. odorifera* rhizospheres in N-supplied soil or N-omitted potting mix, respectively; N + N and N–N: *D. odorifera* nodules in N-supplied soil or N-omitted potting mix, respectively; CK, no inoculation; H255, HT221, and H022238, inoculation with *Bradyrhizobium elkanii* H255, *Rhizobium multihospitium–*like HT221, and *Burkholderia pyrrocinia–*like H022238, respectively.

The distribution of phyla and genera in the nodules and rhizospheres subjected to the eight treatment combinations are presented in Figure 2. Ten phyla, i.e., *Acidobacteria, Actinobacteria, Armatimonadetes, Bacteroidetes, Chloroflexi, Cyanobacteria, Firmicutes, Planctomycetes, Proteobacteria*, and *Verrucomicrobia*, and 32 genera were identified. The genera were *Acetobacter, Acinetobacter, Aquicella, Armatimonas, Arthrobacter, Bacillus, Bradyrhizobium, Caulobacter, Chloroplast* norank, *Comamonas, Devosia, Dyella, Enhydrobacter, Escherichia, Gaiellales* norank, *Lactococcus, Lysinibacillus, Mesorhizobium, Mucilaginibacter, Mycobacterium, Opitutus, Phenylobacterium, Piscinibacter, Planctomyces, Pseudomonas, Psychrobacter, Reyranella, Rhodocyclus, Shigella, Solibacillus, Streptococcus*, and *Xanthomonas*.

The relative abundances of three abundant bacterial phyla (*Firmicutes, Proteobacteria*, and *Cyanobacteria*) differed significantly between the rhizosphere and nodule samples. *Firmicutes* were significantly enriched in the rhizospheres (relative abundance, 46–73%) compared to the corresponding nodules (2–45%). In contrast, *Proteobacteria* and *Cyanobacteria* were significantly enriched in the nodules (21–68% and 9–68%, respectively) compared to the corresponding rhizospheres (14–30% and 1–12%, respectively).

Overall, the most abundant genus in the rhizosphere was *Lactococcus* (31–56% of total sequences for N+ and 25–41% for N−). The *Bradyrhizobium* genus was dominant in the N+ nodules, corresponding to 51% and 60% of total sequences in the N+ *Bradyrhizobium* and N+ *Burkholderia* nodules, respectively. The *Chloroplast* norank group was dominant in N+ *Rhizobium* nodules (44%), and it was also the most prevalent group in the N− nodules, corresponding to 68, 44, 34, and 41% in the *Bradyrhizobium, Rhizobium, Burkholderia*, and uninoculated nodules, respectively. In addition, the *Lactococcus* genus was always enriched in the N+ uninoculated nodules.

Interestingly, three α-rhizobia (*Bradyrhizobium, Mesorhizobium*, and *Rhizobium*) and two β-rhizobia (*Burkholderia* and *Cupriavidus*) were detected in most of the rhizosphere and nodule samples. The exceptions involved *Cupriavidus*, which was absent from the nodules in the N+ media inoculated with *Bradyrhizobium* and *Rhizobium* conditions, and the N− media inoculated with *Burkholderia* condition; and *Rhizobium*, which was absent from the rhizospheres in the N+ media inoculated with *Bradyrhizobium* condition.

At the species level, the non-rhizobial *Lactococcus piscium* was the dominant species in the rhizosphere, regardless of media status. Overall, in the 32 samples, the most abundant rhizobia species was *Bradyrhizobium elkanii*, with a relative abundance of 0.5–1.3% and 20–60% in the N+ rhizosphere and nodules, respectively, and 0.02–0.12% and 12–40% in the N− rhizosphere and nodules, respectively (Table 4). Unclassified *Rhizobium* and *Mesorhizobium plurifarium* were also found in each rhizosphere sample (0.02–0.11%) and nodule sample (0.03–3.41%), with the exception that no Unclassified *Rhizobium* was detected in the N+ *Bradyrhizobium* rhizospheres (Table 4). However, Unclassified *Bradyrhizobium* was only found in the nodules (0.01–0.03%). *Rhizobium huautlense* was detected in both N− *Bradyrhizobium* nodules and rhizospheres (≤ 0.03% for both). *Paraburkholderia mimosarum* was found in all N+ rhizospheres (0.01–0.04%), and in the N+ nodules inoculated with *Rhizobium* and *Burkholderia* (0.01 and 0.04%, respectively) (Table 4). Nevertheless, these rhizobia (including Unclassified *Bradyrhizobium* and *Rhizobium, Mesorhizobium plurifarium* and *P. mimosarum*) were not dominant species in all nodules. A species of *Cyanobacteria*, which was previously isolated from *Phaseolus acutifolius* (the Tepary bean), was the dominant species in all the N− nodules (34–68%).

**Table 4:** Relative abundance of common rhizobia detected in the rhizosphere and root nodule samples of *D. odorifera* seedlings.

**Treatments**	**Relative abundance (%)**					
	***B. elkanii***	**Unclassified *Bradyrhizobium***	***M. plurifarium***	***R. huautlense***	**Unclassified *Rhizobium***	P. mimosarum^*^
***Bradyrhizobium elkanii*** **H255**						
N+ nodule	51	0.02	0.01	–	0.10	–
N+ rhizosphere	0.60	–	0.07	–	–	0.02
N− nodule	12	0.01	0.02	0.01	0.40	–
N− rhizosphere	0.12	–	0.05	0.01	0.04	–
***Rhizobium multihospitium*****-like HT221**						
N+ nodule	33	0.01	0.01	–	0.03	0.01
N+ rhizosphere	1.34	–	0.21	–	0.11	0.02
N− nodule	40	–	0.46	0.12	3.41	–
N− rhizosphere	0.02	–	0.02	–	0.08	–
***Burkholderia pyrrocinia*****–like H022238**						
N+ nodule	60	0.03	0.17	–	0.22	0.04
N+ rhizosphere	0.89	–	0.10	–	0.02	0.04
N− nodule	14	0.01	0.18	0.02	0.73	–
N− rhizosphere	0.02	–	0.05	0.01	0.05	–
**No inoculation**						
N+ nodule	20	0.02	0.14	–	0.13	–
N+ rhizosphere	0.49	–	0.05	–	0.04	0.01
N− nodule	21	0.01	0.35	0.01	1.19	–
N− rhizosphere	0.02	–	0.03	–	0.03	–

*Data were obtained via analyzing the mean OTU value of two replicates of each treatment*.

* *Paraburkholeria mimosarum has been transferred from genus Burkholderia to Paraburkholderia (Oren and Garrity, 2015)*.

### OTU-level analysis

Across all 32 samples, 28 OTUs and 125,564 reads were shared, at a 3% genetic distance (Table 5). The N+ rhizosphere samples shared the greatest number of OTUs (356), most of which belonged to the genus *Lactococcus* (Table 5). Among the N− rhizosphere samples, 221 OTUs were shared. Among the N+ and N− nodule samples, 96 and 103 OTUs were shared, respectively (Table 5).

**Table 5:** The number of shared OTUs and sequences across different samples at a 3% genetic distance generated using a shared OTU table.

**Samples compared**	**OTUs shared**	**Shared sequences**
N+ rhizosphere	356	37,929
N− rhizosphere	221	37,541
N+ nodule	96	39,763
N− nodule	103	38,045
*Bradyrhizobium elkanii* H255	68	37,149
*Rhizobium multihospitium*-like HT221	64	31,514
*Burkholderia pyrrocinia*–like H022238	69	33,158
No inoculation	80	36,505
All samples	28	125,564

*Data were obtained via analyzing the mean OTU value of two replicates of each treatment*.

Regarding inoculation, the uninoculated group shared 80 OTUs, which was higher than the three inoculated groups (Table 5). The *Bradyrhizobium, Rhizobium*, and *Burkholderia* groups shared 68, 64, and 69 OTUs, with 37,149, 31,514, and 33,158 sequences, respectively (Table 5).

**Figure 3:**
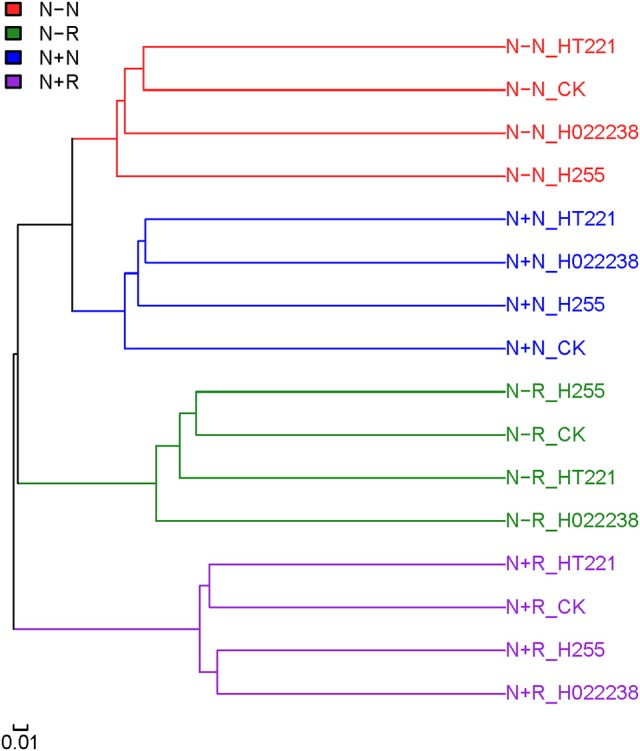
Clustering of rhizosphere and nodule samples of *D. odorifera* seedlings. Based on the abundance of operational taxonomic units (OTUs), an unweighted Unifrac test was performed using QIIME software to verify the sample structure via clustering. Refer to Figure 2 for treatment combination details.

The clustering of the 32 samples was evaluated using unweighted Unifrac (Figure 3). The rhizosphere and nodule samples could be separated clearly. Regardless of rhizobial inoculation or media status, the different treatment combination groups of rhizospheres and nodules were each clustered independently.

### PCA

**Figure 4:**
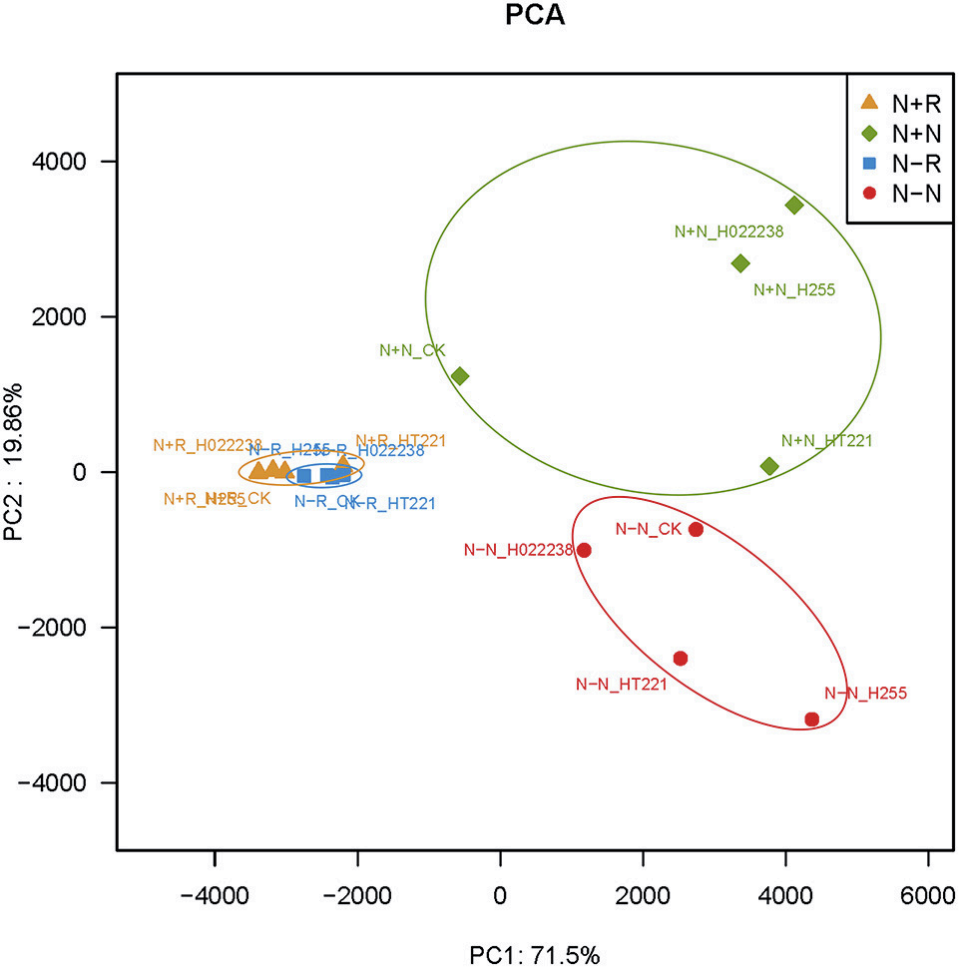
Principal components analysis of operational taxonomic unit (OUT) abundance data for the rhizosphere and nodules of *D. odorifera* seedlings. Refer to Figure 2 for treatment combination details.

The PCA of the 16 treatment combinations showed that the first principal component explained 71.5% of the variance in the OTU data (Figure 4). The PCA demonstrated that the treatment combination-specific bacterial samples could be separated using the OTU data, indicating that the 16 treatment combinations produced significantly different bacterial compositions. Specifically, according to the PCA, OTU4279 (*Lactococcus*) and OTU1501 (*Bacillus*) were enriched in the rhizospheres, while OTU1702 (*Chloroplast* norank) and OTU898 (*Bradyrhizobium*) were more abundant in the nodules (Figure 4).

## Discussion

Our study used a high-throughput sequencing approach to determine the bacterial community compositions in the rhizosphere and root nodules of *D. odorifera* seedlings under four inoculation conditions with two growth media. The experiment permitted us to examine the influence of rhizobial inoculation on bacterial communities in rhizospheres and root nodules under greenhouse conditions. Due to the small nodule number, the nodule samples were not sufficient for the DNA extraction-related analyses. We only had two replicates from 6 plants in each treatment condition for the high-throughput sequencing analysis, and so our data must be considered with caution. However, this process allowed us to assess rhizobial biodiversity associated with legume–rhizobium mutualism.

### Effect of rhizobial inoculation on nodulation and N_2_ fixation

To assess the influence of rhizobial inoculation on nodulation and N_2_ fixation in *D. odorifera* seedlings, this study used three rhizobial strains as inocula, which led to enhanced N_2_ fixation efficiency compared with the uninoculated condition (Table 2). The seedlings inoculated with *Bradyrhizobium* had significantly more nodules than those inoculated with the other two inocula (Table 2). *Bradyrhizobium* has been reported to be an efficient microsymbiont with respect to most legumes, and it is widely acknowledged to be an ecologically and economically important organism (Parker, 2003; Rasolomampianina et al., 2005; Yao et al., 2014; VanInsberghe et al., 2015; Sprent et al., 2017).

The N− potting mix condition induced more nodules than the N+ soil condition (Table 2). Soil N concentration together with other soil properties, including pH and texture, played a vital role in influencing nodulation in this study. Previous research has revealed that high rates of rhizobial nodulation and N_2_ fixation occur in plants in low-N environments (Menge et al., 2015), and the N concentration is known to have a major impact on the symbiosis between rhizobia and host plants. In addition, soil pH could be an important factor influencing legume nodulation and N_2_ fixation (Zahran, 1999). Most legumes require neutral or slightly acidic soil for growth. This is because soil acidity limits rhizobial sustainability, and ultimately their survival, in soils, and thus it reduces nodulation, constrains symbiotic N_2_ fixation, and even shifts gene expression related to various cellular functions in the plant host (Hellweg et al., 2009; Ferguson et al., 2013; Abd-Alla et al., 2014). In the present study, we assume that the media pH could be the determinant factor that influenced nodulation in this pot study, although different media and N levels may also have had some effects on nodulation. Nevertheless, the influences of N status are also worthy of further study.

The ^15^N natural abundance method is an effective approach for estimating N_2_ fixation by potted plants and in agroforestry ecosystems (Unkovich et al., 2008; Denton et al., 2013; Peoples et al., 2015). In our study, 45–57% of the tissue N in the 8-month-old nodulated *D. odorifera* seedlings was derived from N_2_ fixation (Table 2), with the rest being derived from the seeds and growth media, as previously described by Unkovich et al. (2008). These N_2_ fixation rates are comparable to those calculated in our previous study, in which 41–44% of tissue N in 6-month-old *D. odorifera* seedlings was derived from fixation (Lu et al., 2013).

### Effects of rhizobial inoculation on bacterial communities

In general, microbial inoculation can change, at least temporarily, the native bacterial community in the soil (Schlaeppi and Bulgarelli, 2015). Bacterial communities in the soil consist of a complex mix of endemic and newly introduced taxa, with a degree of genetic exchange between rhizobia and other soil bacteria (Nandasena et al., 2007). Our study showed that rhizobial inoculation altered the bacterial communities in the rhizosphere and nodules regardless of growth media status, with a particularly high Shannon index for nodules in the N+ inoculated conditions (Table S3). Differences in the physiology of bacteria (including their ability to establish diverse bacteria–bacteria interactions) influence the composition of microbial communities in the soil, rhizosphere, and legume nodules (Masson-Boivin et al., 2009; Peiffer et al., 2013; Peix et al., 2015; Shi et al., 2015). Denton et al. (2013) demonstrated that inoculation of faba beans with sufficient rhizobia can increase N_2_ fixation in the faba beans in the presence of massive quantities of soil rhizobia, suggesting that inoculant rhizobia and soil bacteria might form an association for effective nodulation and N_2_ fixation. However, little is known about the buffering capacity of the soil and plant microbiota. Hence, understanding the persistence of inoculated bacteria in the field and their effects on the native microbial communities has theoretical and practical significance with respect to the use of microbial inoculants in agroforestry.

### Bacterial diversity in the rhizosphere

Microbes in the rhizosphere play key roles in the growth and ecological fitness of host plants. Common physiological processes exhibited by these microbes include phytopathogenic activity, phytohormone production, atmospheric N_2_ fixation, geochemical mineral cycling, and plant colonization (Buée et al., 2009; Peiffer et al., 2013). Members of the phyla *Firmicutes* and *Proteobacteria* are well-known rhizosphere colonizers and have generally been characterized as fast-growing *r*-strategists that respond positively to plant root exudates (e.g., low-molecular-weight substrates) (Shi et al., 2015). This is consistent with our results, which showed that a few taxa were consistently enriched in the rhizosphere of *D. odorifera* regardless of media status, such as the phyla *Firmicutes* and *Proteobacteria*, and the genera *Lactococcus, Bacillus*, and *Pseudomonas*. The genus *Lactococcus* has been shown to be enriched in the rhizosphere, seed and rice silage (Ennahar et al., 2003; Fhoula et al., 2013; Adam et al., 2016), and bacteria in this genus are known to produce biological control agents (see Adam et al., 2016). Recent studies of lettuce and sweet potato have also noted enrichment of *Bacillus* and *Pseudomonas* in the rhizosphere (Marques et al., 2014; Schreiter et al., 2014), both of which play an important role in plant health by suppressing plant pathogens (Santoyo et al., 2012).

### Coexistence of rhizobia and non-rhizobial bacteria in nodules

Using a traditional cultivation method, our recent research verified that *D. odorifera* can establish N_2_-fixing symbiosis with *Bradyrhizobium, Ensifer, Rhizobium*, and *Burkholderia* (Lu et al., 2011, 2012), suggesting that *D. odorifera* can efficiently establish symbioses with a wide range of rhizobial species. A study by Rasolomampianina et al. (2005) also showed that several *Dalbergia* species endemic in Madagascar were able to form symbioses with bacteria in the genera *Azorhizobium, Bradyrhizobium, Mesorhizobium, Rhizobium*, and *Phyllobacterium* (which belong to the *Alphaproteobacteria* class), as well as with bacteria in the genera *Burkholderia* and *Ralstonia* (which belong to the *Betaproteobacteria* class) (Rasolomampianina et al., 2005). In the present study, the genera enriched in the nodules included *Bradyrhizobium, Chloroplast* norank (belonging to *Cyanobacteria*), *Lactococcus, Mycobacterium*, and *Bacillus*, whereas common rhizobia genera, such as *Rhizobium, Mesorhizobium*, and *Burkholderia* were found at low relative abundances in the nodules (Figure 2). This study, which relied on a metagenomic approach, provides the first report on the full diversity of bacteria in *D. odorifera* nodules, especially regarding large relative abundance of *Cyanobacteria*.

Legume nodules represent a distinctive ecological niche, with an adapted program for the accommodation of compatible soil microbes. Apart from rhizobia, legume nodules are often occupied by a phylogenetically diverse bacterial microbiome. These co-evolved bacteria may impact plant growth and health or even nodule formation and N_2_ fixation, but their ecological roles remain unknown (Bai et al., 2003; Sachs and Simms, 2008; Buée et al., 2009; Peix et al., 2015). Although the bacterial diversity in root nodules was significantly lower than that in the corresponding rhizospheres, the species diversity and relative abundance of non-rhizobial bacteria in the nodules were greater than expected. The most abundant rhizobia were distributed among five genera, with *Bradyrhizobium* being the predominant genus. In addition to the abovementioned rhizobia, 32 genera of non-rhizobial bacteria were found in all the *D. odorifera* nodule samples. These results indicate the coexistence of diverse non-rhizobial bacteria with rhizobia in the *D. odorifera* nodules in this study.

Non-rhizobial bacteria are considered to be competent to infect and multiply within legume nodules. However, it has been assumed that rhizobia symbionts dominate the interior of the nodules, with an adapted ability to outcompete other bacteria and to efficiently communicate with the host during infection (Zgadzaj et al., 2015). In contrast, Sachs and Simms (2008) found that non-rhizobial bacteria may dominate, encompassing up to 99% of the total bacterial population in legume nodules. In our study, all the nodules of the N- samples contained non-rhizobial bacteria as the predominant population (56–87% of the total bacterial population), while nearly half of the bacterial population in the nodules of the N+ samples were non-rhizobial bacteria. The ubiquity of non-rhizobia taxa raises the question of whether they exploit the opportunity to invade nodules as an adaptive strategy. Some researchers believe that the non-rhizobial bacteria might invade nodules that are formed by nodulating rhizobia (Wu et al., 2011; Mus et al., 2016), which have improved fitness compared to other soil bacteria or a better ability to communicate with the legume host (Zgadzaj et al., 2015). However, recent studies have shown that non-rhizobial bacteria in nodules are not passive players (Zgadzaj et al., 2015; Gano-Cohen et al., 2016). They may even control the host plant to some degree, using exopolysaccharides to subtly influence the host during their chronic infection of the nodules (Zgadzaj et al., 2015). In addition, some non-rhizobial bacteria help rhizobium to extend their host range (Liu et al., 2010) and improve the nodulation and N_2_ fixation of legume–rhizobia symbionts (Peix et al., 2015). In contrast to these positive effects, some non-rhizobial bacteria may be able to reduce the fitness of nodulating rhizobia via competitive exclusion in the rhizosphere (Gano-Cohen et al., 2016).

## Author contributions

All authors listed have made a substantial, direct and intellectual contribution to the work, and approved it for publication.

### Conflict of interest statement

The authors declare that the research was conducted in the absence of any commercial or financial relationships that could be construed as a potential conflict of interest.
