# Supplementary figures and images for: Co-existence of Rhizobia and Diverse Non-rhizobial Bacteria in the Rhizosphere and Nodules of Dalbergia odorifera Seedlings Inoculated with Bradyrhizobium elkanii, Rhizobium multihospitium–Like and Burkholderia pyrrocinia–Like Strains
Source: Front Microbiol. 2017 Nov 21;8:2255. doi: 10.3389/fmicb.2017.02255 (PMC5702347; doi:10.3389/fmicb.2017.02255)

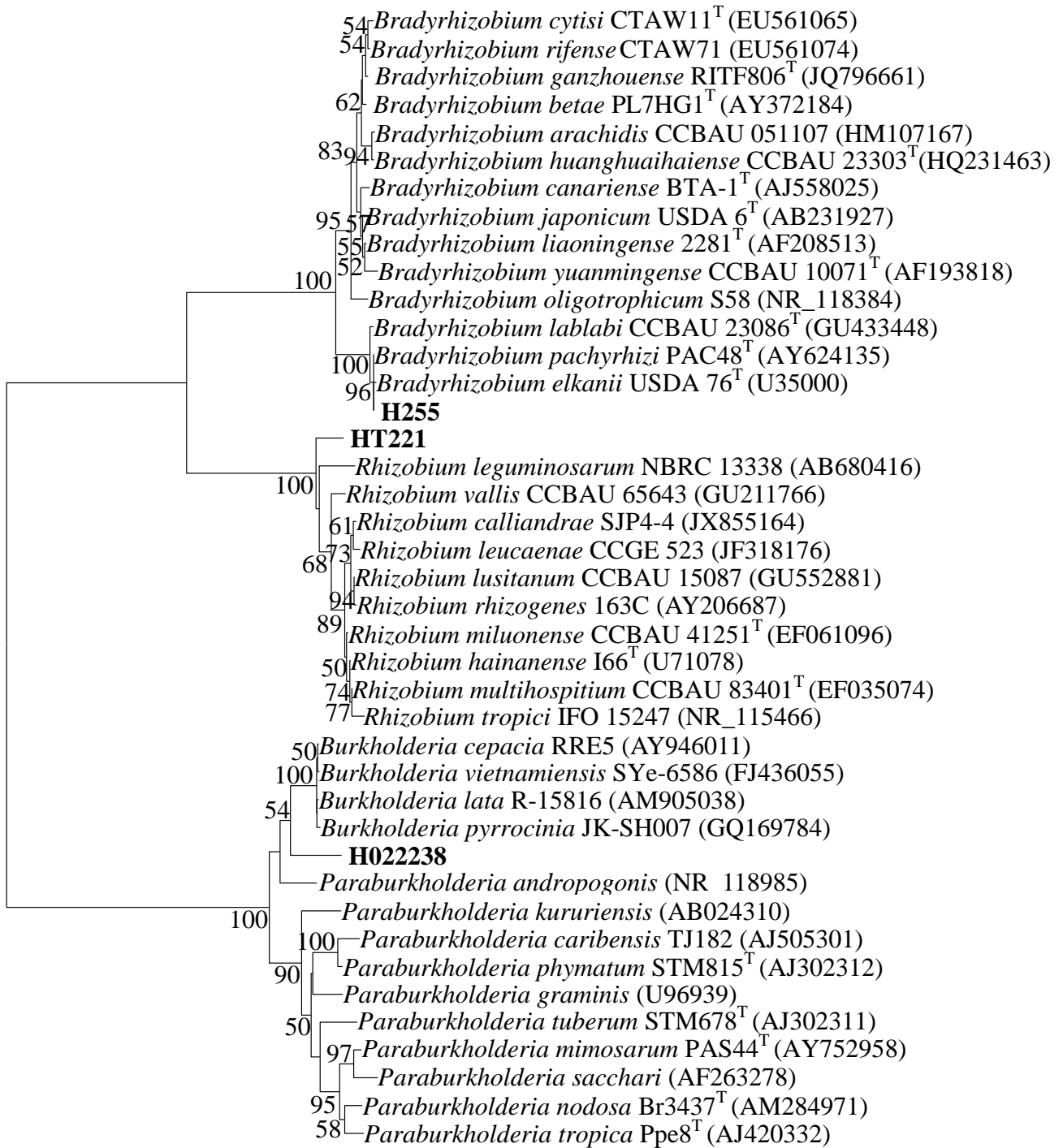

0.02

Supplement: Supplementary file 1 [file Image1.PDF]

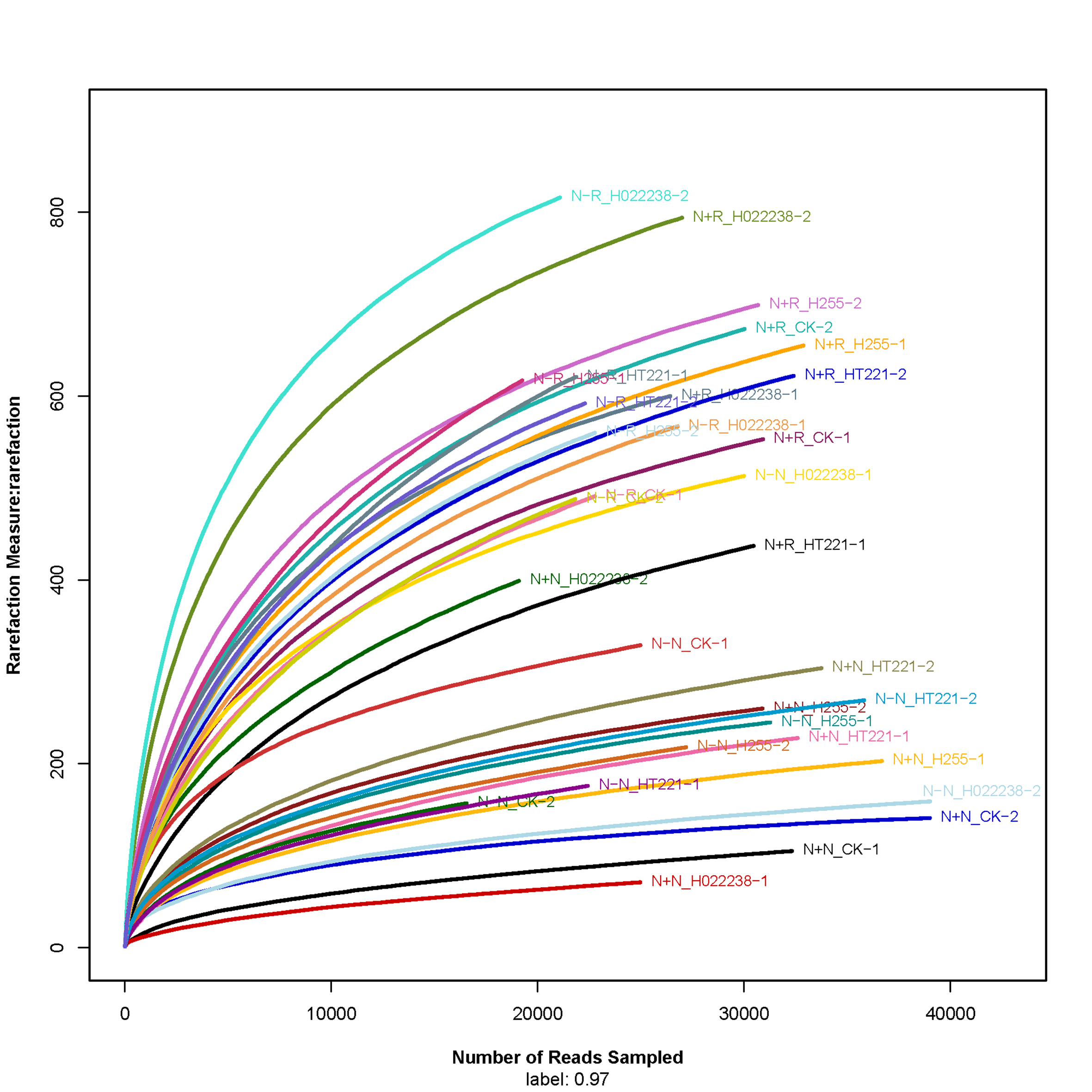

Supplement: Supplementary file 2 [file Image2.TIF]
