# Supplementary material for: Co-existence of Rhizobia and Diverse Non-rhizobial Bacteria in the Rhizosphere and Nodules of Dalbergia odorifera Seedlings Inoculated with Bradyrhizobium elkanii, Rhizobium multihospitium–Like and Burkholderia pyrrocinia–Like Strains
Source: Front Microbiol. 2017 Nov 21;8:2255. doi: 10.3389/fmicb.2017.02255 (PMC5702347; doi:10.3389/fmicb.2017.02255)
Supplement: Supplementary file 3 [file Table1.doc]

**SUPPLEMENTARY MATERIALS**

**FIGURE LEGENDS**

FIGURE S1 Neighbour-joining tree reconstructed with the Kimura two-parameter model of 16S rRNA gene sequences showing the relationships among strains H255, HT221 and H022238 and the type strains of some recognized species. Bootstrap confidence levels are indicated at the nodes. Bar, 0.02 expected changes per site.

FIGURE S2 Rarefaction curves for the numbers of observed OTUs defined by no more than 3% difference between 16S rRNA gene sequences. Refer to Fig. 2 for treatment details.

**TABLE**

**TABLE S1**

Results of two-way ANOVA for the effects of rhizobia inoculation (I) and nitrogen supply (N) on nodule number, foliar δ^15^N and N concentration

|  | Nodule number | Foliar δ^15^N | N concentration |
| --- | --- | --- | --- |
| I | **63.3** | **32.8** | **1.03** |
|  | *26450* | *37.2* | *0.613* |
|  | <0.001 | <0.001 | 0.388 |
| N | **194** | **35.5** | **153** |
|  | *26980* | *13.4* | *30.3* |
|  | <0.001 | <0.001 | <0.001 |
| I × N | **55.8** | **4.11** | **1.55** |
|  | *23312* | *4.66* | *0.919* |
|  | <0.001 | 0.012 | 0.217 |

*F*, sum of square and *P* values are in bold, italic and regular type, respectively.

**TABLE S2**

Results of one-way ANOVA for the effects of rhizobia inoculation (I) on nitrogen fixation efficiency and specific nodule activity

|  | Nitrogen fixation efficiency | Specific nodule activity |
| --- | --- | --- |
| I | **3.18** | **4.40** |
|  | *428* | *39678* |
|  | 0.046 | 0.016 |

*F*, sum of square and *P* values are in bold, italic and regular type, respectively.

**TABLE S3**

Results of Adonis analysis for the OTU data between treatments for a given sample and between samples for a given treatment

| Samples compared | *F* | Sum of square | *P* |
| --- | --- | --- | --- |
| N+ rhizosphere (*n*=8) | 8.10 | 1.00 | 0.103 |
| N- rhizosphere (*n*=8) | 2.20 | 0.26 | 0.014 |
| N+ nodule (*n*=8) | 1.75 | 0.49 | 0.008 |
| N- nodule (*n*=8) | 2.00 | 0.33 | 0.044 |
| *Bradyrhizobium* H255 (*n*=8) | 33.3 | 1.74 | 0.009 |
| *Rhizobium* HT221 (*n*=8) | 9.09 | 2.06 | 0.017 |
| *Burkholderira* H022238 (*n*=8) | 9.61 | 1.42 | 0.020 |
| Non inoculation (*n*=8) | 3.48 | 1.18 | 0.034 |
| All 32 samples | 7.39 | 7.06 | 0.001 |
